# Evaluation of Child–Computer Interaction Using Fitts’ Law: A Comparison between a Standard Computer Mouse and a Head Mouse

**DOI:** 10.3390/s21113826

**Published:** 2021-05-31

**Authors:** Cristina Sanchez, Vanina Costa, Rodrigo Garcia-Carmona, Eloy Urendes, Javier Tejedor, Rafael Raya

**Affiliations:** Departamento de Tecnologías de la Información, Escuela Politécnica Superior, Universidad San Pablo-CEU, CEU Universities, 28668 Madrid, Spain; vanina.costacortez@ceu.es (V.C.); rodrigo.garciacarmona@ceu.es (R.G.-C.); eloyjose.urendesjimenez@ceu.es (E.U.); javier.tejedornoguerales@ceu.es (J.T.); rafael.rayalopez@ceu.es (R.R.)

**Keywords:** child–computer interaction, augmentative and alternative communication, Fitts’ law, throughput, human motor performance, psychomotor development, computer mouse, head mouse, inertial sensor, learning process, pointing task

## Abstract

This study evaluates and compares the suitability for child–computer interaction (CCI, the branch within human–computer interaction focused on interactive computer systems for children) of two devices: a standard computer mouse and the ENLAZA interface, a head mouse that measures the user’s head posture using an inertial sensor. A multidirectional pointing task was used to assess the motor performance and the users’ ability to learn such a task. The evaluation was based on the interpretation of the metrics derived from Fitts’ law. Ten children aged between 6 and 8 participated in this study. Participants performed a series of pre- and post-training tests for both input devices. After the experiments, data were analyzed and statistically compared. The results show that Fitts’ law can be used to detect changes in the learning process and assess the level of psychomotor development (by comparing the performance of adults and children). In addition, meaningful differences between the fine motor control (hand) and the gross motor control (head) were found by comparing the results of the interaction using the two devices. These findings suggest that Fitts’ law metrics offer a reliable and objective way of measuring the progress of physical training or therapy.

## 1. Introduction

Child–computer interaction (CCI) is the branch within human–computer interaction (HCI) that studies the design, implementation and use of interactive computer systems for children [1]. The research on this area has gained traction since the 1990s, mainly driven by interest in the use of technology in schools, for educational and communication purposes. An essential goal when designing human–computer interfaces for children is to make them “child friendly”, by developing interactions designed to provide a natural feel and the sensation of control to children. These design requirements are even more relevant when the interfaces are focused on children with special needs.

Augmentative and alternative communication (AAC) is the field that studies hardware and software resources that bridge the gap between computers or personal devices and people with disabilities. Cerebral palsy (CP) is the most common physical disability in childhood, affecting 2–3 per 1000 live births [2]. CP is an umbrella term that describes motor disorders caused by a lesion in the immature brain [3]. These disorders, which affect movement and posture, are often accompanied by cognitive or perceptive disorders that greatly hamper daily life activities, limiting the capabilities for communication and social relationships among children. According to population-based studies, one out of two children with CP have a speech disorder [4]. Therefore, alternative and augmentative interfaces could give such children the opportunity to improve their interaction with their physical and social environment.

Children with CP often use alternative and augmentative input devices as substitutes to a standard computer mouse; trackballs, mechanical switches, joysticks or adapted keyboards are the most common devices [5]. Recently, touchscreen tablets have introduced a very cost-effective human–computer interface for children with motor disorders [6]. Other emerging technologies, such as gaze trackers or head mice, have become popular as a solution for many users with CP [7].

The ENLAZA interface is an alternative input device that replaces a standard computer mouse. It is based on a wearable inertial sensor that measures the head posture, which is translated into mouse pointer movements. This interface has been previously used for different purposes: input device [8], physical rehabilitation [9], and biomechanical assessment [10]. One of the first works that analyzed the ENLAZA interface was Fitts’ law, which extracted the throughput (*TP*) for healthy adult subjects [11].

Fitts’ law (1954) is the first mathematical model that describes the trade-off between speed and accuracy during reaching tasks [12]. It is defined as shown in Equation (1):

(1) $$ID=log_{2}\left(\frac{A}{W}+1\right)$$

where *ID* is the index of difficulty, and *A* and *W* are the distance between targets (measured from the center of the targets) and the width of each target, respectively.

This law was later adapted according to the Shannon formulation by Mackenzie [13], and was improved regarding its accuracy following Crossman’s research [14], giving rise to the current model. The main metric, the *TP*, is calculated as Equation (2):

(2) $$TP=\;\frac{ID_{e}}{MT}$$

where *MT* is the movement time average over a sequence of trials in seconds (s), and *ID_e_* is the effective index of difficulty of the selected task in bits and is obtained according to Equation (3):

(3) $$ID_{e}=log_{2}\left(\frac{A}{W_{e}}+1\right)$$

where *W_e_* is the effective target width (replaces *W* from the original formulation of Fitts’ law), and is calculated from the standard deviation (*SD*) in the selection coordinates gathered over a sequence of trials for a particular *A-W* condition, as shown in Equation (4):

(4) $$W_{e}=4.133\cdot SD$$

In addition, the linear relationship between *MT* and *ID_e_* can be expressed as stated in Equation (5):

(5) $$MT=a+b\cdot ID_{e}$$

where *a* and *b* are constants that depend on the choice of the input device and are usually determined empirically by regression analysis. Constant *a* defines the intersection on the *y* axis and can be interpreted as a delay. Constant *b* is a slope describing the acceleration. Both parameters show the linear dependency in Fitts’ law.

Finally, it is important to highlight that the index of performance (*IP*), defined as Equation (6):

(6) $$IP=\frac{1}{b}$$

which allows for the comparison of different pointing devices; the higher its value, the less *MT* is affected by increases in *ID_e_*.

Over the last 30 years, Fitts’ law and its subsequent adaptations have been widely used in HCI to evaluate the performance of input devices [13], as well as in other research fields, including kinematics and motor behavior [15,16].

In the field of HCI, the standard ISO 9241-9 “Ergonomic requirements for office work with visual display terminals (VDTs)—Part 9: Requirements for non-keyboard input devices” (revised by ISO 9241-400:2007) [17], indicates that the *TP* is a useful factor to assess the usability of input devices. In fact, this metric has been widely used to evaluate alternative input devices. Bernardos et al. [18] obtained a *TP* of 2.04 bits/s for a head-based interaction using a Kinect device. Roig et al. [19] evaluated a system based on head tracking using a mobile device’s front camera, obtaining a *TP* of 1.42 bits/s. Raya et al. [20] studied the *TP* of the ENLAZA device, obtaining a value of 1.8 ± 0.4 bits/s. These results agree with those presented in other works in the literature [13,21,22]. In all of these studies, the participants were adult people.

In the case of children, Fitts’ law has mainly been used to investigate how children control their movements [23]. The proposed task usually consists of performing rapid pointing movements between two targets with different combinations of distances and widths (which determine the index of difficulty of the task). These tasks can be performed in both the real world or a simulated one, which is why Fitts’ law is useful for evaluating motor behavior and HCI. In both scenarios, Fitts’ law defines a linear relation between *MT* and *ID_e_*, whose slope usually quantifies the amount of information processed per second. Recent studies have investigated the user experience of children using touchscreen tablets, reporting a *TP* of around 2.3–2.5 bits/s [24], and complemented these findings with some recommendations for professionals who use these devices for teaching [25].

In the field of human behavior, it has been proven that Fitts’ law can be used to evaluate the maturity of the central nervous system (CNS), whose role is in processing the information needed for motor skills [26,27], leading the motor system. The quicker this information is processed, the better the movements can be executed. Therefore, measuring the time and spatial accuracy of movements together provides relevant information about the development of the sensorimotor control. Hertzum et al. [28] studied the effects of age on pointing performance with mouse and touchscreens in groups of young people (12–14 years), adults (25–33) and elderly people (61–69). They showed that adult participants performed better than both young and elderly participants, but they did not provide the *TP* for every group.

Regarding the use among people with disabilities, Gump et al. [29] applied Fitts’ law to individuals with CP to characterize motor behavior during aiming tasks, and Bertucco et al. [30] characterized dystonia of children with CP in reaching tasks using an iPad^TM^. Hay et al. [23] demonstrated that the trade-off between speed and accuracy improves as children grow up, based on analysis of children aged 5 to 11. Schneiberg et al. [31] concluded that children between 8 and 10 years old had outcome measures similar to adults, whereas younger children showed immature patterns during reaching tasks.

The results arising from the aforementioned studies suggest that ages ranged between 0 and 8 years old deserve special attention when designing and using HCIs.

The objective of this study is to evaluate two CCI devices, a standard computer mouse versus the ENLAZA interface, by measuring the motor performance and the learning process of children in early stages of fine motor development performing a multidirectional pointing task. This evaluation is based on the interpretation of Fitts’ law derived metrics: *TP* (mainly), *MT* and *IP*. Despite the computer mouse being the gold standard of the HCI, only a few studies [32] measured the *TP* when users are children. According to the previously cited literature, the slope of the linear equation of MT versus *ID_e_* quantifies the amount of information processed per second. For this study, we found that the linear relation between *MT* and *ID_e_* is maintained, but we also obtained meaningful differences between those slopes for hand control and head control, since the former uses fine motor control, and the latter uses gross motor control. Finally, the study explores some conclusions that could be drawn from the metrics associated with Fitts’ law regarding the performance of a pointing task. These conclusions obtained for children can then be compared with those previously published for adults [13,18,19,20,21,22].

## 2. Materials and Methods

This study compares two CCI devices, a standard computer mouse versus the previously described head mouse (ENLAZA interface), by measuring the motor performance and the learning process while performing multidirectional pointing tasks.

### 2.1. Participants

Ten healthy children, who were randomly recruited from among all the students in their first years of primary school from the Colegio CEU Montepríncipe in Madrid, participated in the study, in order to obtain a representative sample. Ages ranged from 6 to 8 years. All the children had previous experience in the use of a standard mouse, but no one had experience in the use of any kind of head mouse. All of the children’s parents and the head office of the school gave consent for participation.

### 2.2. Experimental Setting

Participants sat in a chair in front of a table, where there was a 17-inch screen laptop lying perpendicular, centered in front of their heads. The laptop had a standard mouse connected, which lay on top of the table. The head mouse was fixed to the forehead of the participants thanks to a wide rubber band that firmly held the device to prevent it from moving relative to their heads during each experiment.

All of the participants were asked to sit comfortably but with their back straight, trying not to change their trunk position during the whole experiment. In addition, the room where the experiments took place was brightly lit and quiet enough to prevent participants from becoming distracted during the tasks.

### 2.3. Test

**Figure 1:**
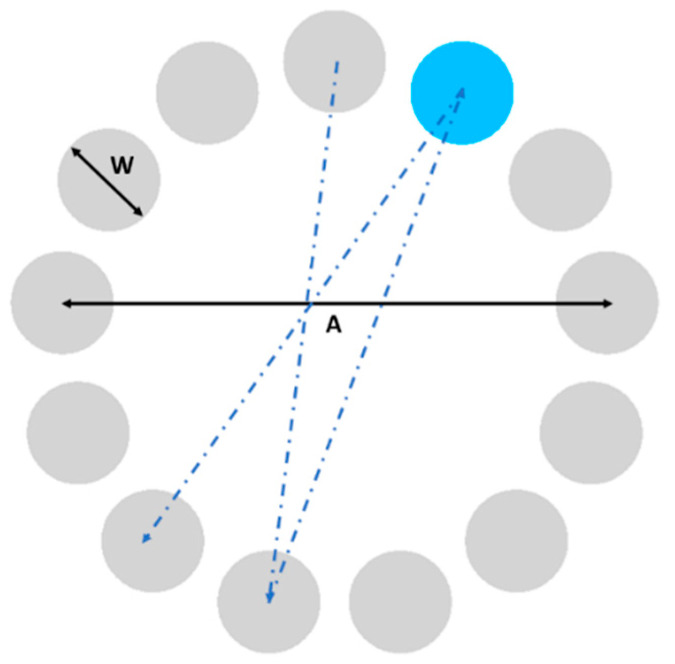
ISO 9241-411 multi directional point-select test (*W*: diameter of each circular target, *A*: amplitude of each movement).

ISO 9241-411 [33] describes a multi-directional test that evaluates point-select actions in different directions through a series of pointing tasks. For these tasks, the user must move the cursor, trying to reach several circular targets (of diameter *W* each) which were equally spaced in a circular layout of diameter *A* (so each movement’s amplitude is *A*) (see Figure 1). The user must start the task with the cursor in the center of the circular layout defined by the set of targets, and the next target will be determined by a change in color (grey to blue), chosen so it can be easily perceived by people with color blindness.

This test, set up and played on the laptop screen using the open source software FittsStudy [34], was performed for a standard mouse and the head mouse, in two consecutive executions. It was randomly determined whether the standard mouse or the head mouse was used first.

Each test displayed a different sequence of 10 targets, randomly determined. The next target to be reached is highlighted and, if the user misses it, i.e., if the target was not correctly selected, the highlight color changes to red. Each sequence (test) was repeated 13 times: the first 3 times for practicing, and the last 10 for testing. This set of 13 sequences were performed 4 times, each with different *A-W* conditions (which led to 4 different indexes of difficulty, *ID*), one after another. The order in which the 4 conditions were chosen was randomly determined (see Table 1).

Participants were asked to select targets as quickly and accurately as possible, but also were told to slow down after more than 3 consecutive targets were missed in the same test. They could rest as much as they wanted between tests.

To study the influence of the learning process when carrying out the described test, each participant’s data were recorded from the first session (called pre-training in the analysis that follows, when the participants had never performed the described test before) to after 3 days of training (called post-training). All participants undertook the same training regime.

### 2.4. Data Analysis and Statistics

After conducting the described experiments, the associated data, previously generated by the software FittsStudy, were exported and analyzed, using both a custom spreadsheet and RStudio software [35].

The variables analyzed after the experiments were *TP*, *MT*, *IP*, and error rate. The analysis was mainly focused on detecting the differences in the users’ performance for each input device (standard mouse and head mouse) and studying the effect of the learning process before and after the training.

First, to test Fitts’ law, the least-squares method was used to carry out linear regression. The bivariate correlation (Pearson correlation coefficient) was also calculated for each case. In addition, *TP*, *MT*, and *IP* were calculated and compared for paired samples of each input device for the pre-training and post-training sessions. On the one hand, the appropriate mean values and their standard deviation were calculated. On the other hand, each comparison started with a series of normality tests on the differences (pre-training and post-training) for both the standard mouse and the head mouse (Shapiro–Wilk test, skew S and kurtosis K standardized parameters, and Q-Q plots with 0.95 confidence intervals). Once the assumption of normality was checked, a *t*-test for paired samples was performed in each case. Moreover, error rate comparisons were performed with a Wilcoxon signed-ranked test for paired samples, as the data were not normally distributed in this case. Finally, the error rate separated for both *W* and *A* values was also calculated and compared for each input device.

## 3. Results

**Figure 2:**
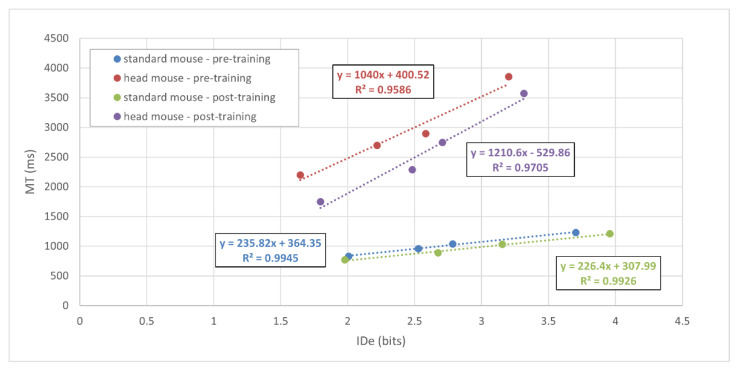
*MT* versus *ID_e_* for all subjects. The dotted lines show the best fits by the least squares’ method for each condition.

Fitts’ law was confirmed for both a standard mouse and the head mouse (see Figure 2). The linear regression equations and the corresponding correlation coefficients (ρ) for the standard mouse were: $y\;=235.82x\;+364.35,{\;R}^{2}=0.99,\;\rho \;=1$ (standard mouse—pre-training, blue dotted line) and $y\;=226.40x\;+307.99,{\;R}^{2}=0.99,\;\rho \;=0.98\;$(standard mouse—post-training, green dotted line). Likewise, the results for the head mouse were: $y\;=1040.00x\;+400.52,{\;R}^{2}=0.96,\;\rho \;=0.97$ (head mouse—pre-training, red dotted line) and $y\;=1210.60x\;-529.86,{\;R}^{2}=0.97,\;\rho \;=0.99$ (head mouse—post-training, purple dotted line).

**Figure 3:**
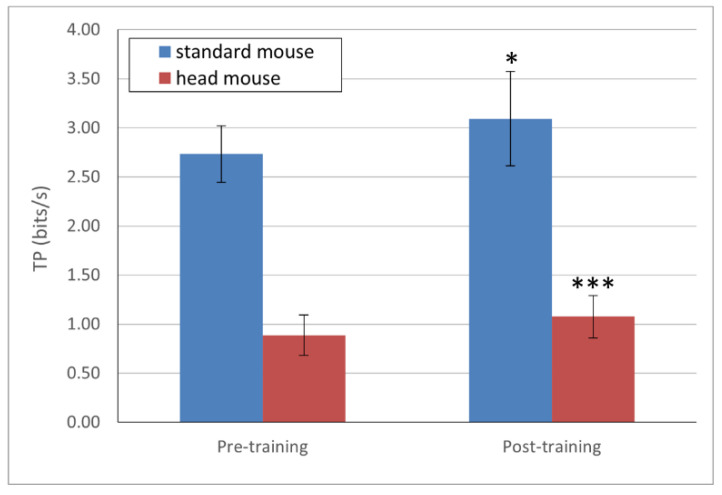
*TP* mean values and standard deviations. Statistically significant changes for both the standard mouse and the head mouse (*p* < 0.05 [*] and *p* < 0.001 [***] respectively) comparing pre- and post-training.

*TP* mean values (in bits/s) and their corresponding standard deviations for each device are presented in Figure 3. For the standard mouse, the results show values of 2.73 ± 0.27 and 3.09 ± 0.48, for pre- and post-training, respectively (blue bars); and 0.89 ± 0.21 and 1.08 ± 0.22 for the head mouse for pre- and post-training, respectively (red bars). The comparison study of the learning effect for *TP* (pre-training vs. post-training) showed a statistically significant result for both the standard mouse (*p* = 0.02) and the head mouse (*p* = 0.0004). The difference was much more remarkable in the case of the head mouse.

**Figure 4:**
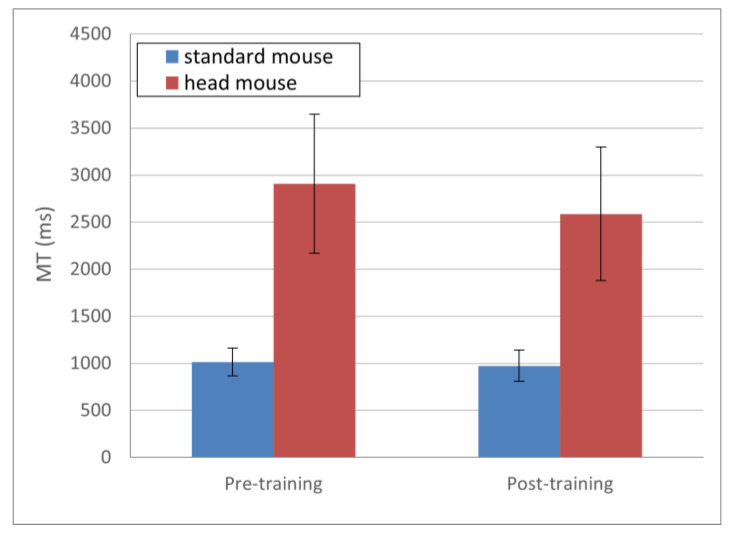
*MT* mean values and standard deviations. No statistically significant changes comparing pre- and post-training (*p* > 0.1 for both the standard mouse and the head mouse).

Regarding the *MT* evolution, presented in Figure 4, the obtained results (in ms) for the standard mouse were 1014.25 ± 146.91 and 973.90 ± 165.52 for pre- and post-training, respectively (blue bars); and 2910.12 ± 738.53 and 2588.83 ± 709.95 for the head mouse for pre- and post-training, respectively (red bars). Although a slightly decreasing tendency in *MT* (greater in the case of the head mouse) can be seen in Figure 4, there were not statistically significant differences, either in the standard mouse (*p* = 0.47) or in the head mouse (*p* = 0.1) comparisons.

**Figure 5:**
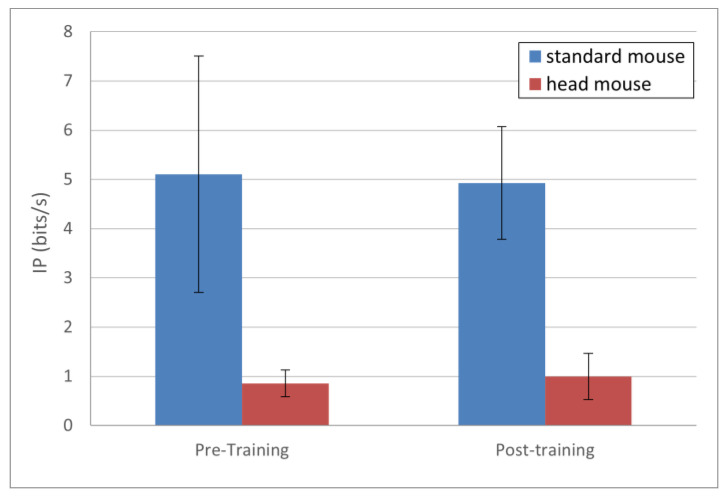
*IP* mean values and standard deviations. No statistically significant changes comparing pre- and post-training (*p* > 0.1 for both the standard mouse and the head mouse).

The results for *IP*, presented in Figure 5, show very few changes in this parameter for both the standard mouse and the head mouse comparing pre- and post-training sessions. Numerically, the results (in bits/s) were as follows: for the standard mouse 5.10 ± 2.40 and 4.93 ± 1.15 for pre- and post-training, respectively (blue bars), and for the head mouse 0.86 ± 0.22 and 0.99 ± 0.47 for pre- and post-training, respectively (red bars). There were not statistically significant differences, either in the standard mouse (*p* = 0.68) or in the head mouse (*p* = 0.35) comparisons.

**Figure 6:**
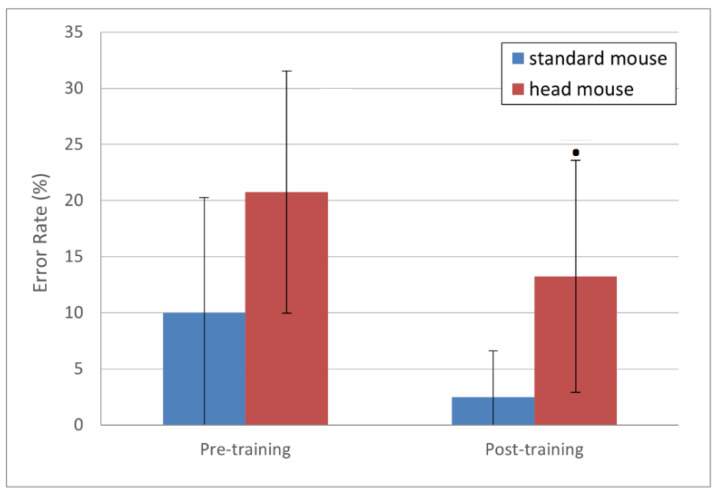
Error rate (%) mean values and standard deviations. Statistically significant changes for the head mouse (*p* < 0.1 [**·**]) (not normally distributed samples, parametric test: Wilcoxon signed rank test with continuity correction with paired samples).

Error rate results, presented in Figure 6, show large standard deviation values, which suggests large data dispersion. Nevertheless, the error rate means (in %) for the standard mouse, 10.00 ± 10.27 and 2.50 ± 4.08 for pre- and post-training, respectively (blue bars), are clearly smaller than those for the head mouse, 20.75 ± 10.80 and 13.25 ± 10.34 for pre- and post-training, respectively (red bars). Regarding the evolution of this parameter for each device comparing pre- and post-training sessions, and although the downward trend is clearly observed, the statistically significant difference only shows for the head mouse (*p* = 0.09 for the head mouse versus *p* = 0.12 for the standard mouse).

**Figure 7:**
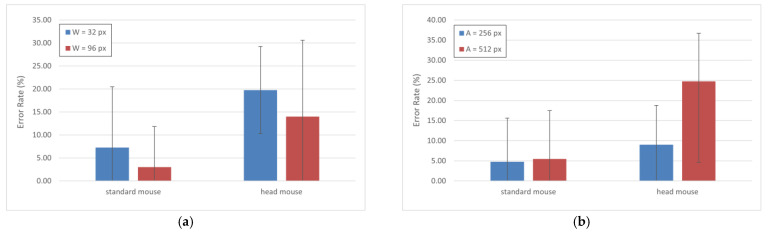
Error rate (%) by (**a**) different values of *W* and (**b**) different values of *A*.

Finally, in Figure 7, we present the comparison of the error rate according to both *W* and *A* values. As expected, these results show that, for both input devices, the error rate is higher as *W* decreases and *A* increases. The percentage of error rate is smaller for the standard mouse in any case, which matches with the results obtained in the previous comparison (see Figure 6). Standard deviations are also remarkably large in this case, and so is the consequent data dispersion. Results (in %) for the standard mouse and the head mouse were: 7.25 ± 13.20 and 19.75 ± 9.49, respectively, for *W* = 32 px (blue bars, Figure 7a), 3.00 ± 8.83 and 14.00 ± 16.61, respectively, for *W* = 96 px (red bars, Figure 7a), 4.75 ± 10.86 and 9.00 ± 9.82, respectively, for *A* = 256 px (blue bars, Figure 7b), and 5.50 ± 11.97 and 24.75 ± 20.13, respectively, for *A* = 512 px (red bars, Figure 7b).

## 4. Discussion

Research on CCI has grown considerably in the last three decades, mainly motivated by interest in educational applications. Different studies state that the usability of computers is strongly dependent on motor skills, which suggests that the children’s needs must be carefully studied and considered. New interactive systems (i.e., touchscreens, tablets, or smartphones) have introduced scenarios with a greater usability, facilitating the use of computers by children.

Over the decades, Fitts’ law has been used to evaluate HCI. However, there are a lack of studies evaluating CCI using Fitts’ law and the tests proposed in the standard ISO 9241. Fitts’ law states that the amount of time required to move a pointer to a target area is a function (linear dependence) of the index of difficulty, which, in turn, depends on the distance to the target and the target size.

Regarding our results, Figure 2 depicts the values of the *MT* (mean values ± standard deviation) versus the *ID_e_* using a standard mouse and the head mouse. The subsequent linear regression adjustment demonstrates that the performance for all conditions follows the described Fitts’ model. Therefore, it can be concluded that the metric proposed by Fitts’ law, the *TP*, is reliable to evaluate the use of a standard mouse and the head mouse by children with ages ranged between 6 and 8.

In addition, Figure 2 also shows that the slopes of the regression lines for the tests using the head mouse are higher than those for a standard mouse. Since the slope of that linear equation usually quantifies the amount of information processed per second, this result makes possible the identification of differences between both motor behavior and control of hand and head. As expected, the former is more precise than the latter. This result suggests that the slope of the linear equation might be a useful metric to evaluate the motor performance of a training regime or physical therapy. Therefore, the *TP* evaluation might complement gross motor functional scales, by enabling the detection of finer changes in motor performance.

According to Gallahue, who deeply studied the psychomotor developmental stages [36], children around 6–7 begin to acquire fine motors skills (fine and precise movements) and it is only around 14, once those fine motor skills are well established, that they begin to develop special skills. This insight could explain why the mean *TP* values obtained for a standard mouse in our experiments (2.7 and 3.1 bits/s for the pre- and post-training sessions, respectively, as shown in Figure 3) are smaller than those expected for healthy adults (from 3.7 to 4.5 bits/s) [21]. An interesting approach for subsequent studies could be to analyze changes in the *TP* values across all age ranges of psychomotor development (as described by Gallahue) since, although the normal ranges for the *TP* parameter are widely studied and established for adults, there is a lack of scientific evidence describing this parameter in children.

However, the obtained mean *TP* values for the head mouse, starting at 0.89 bits/s and climbing to 1 bit/s after the training process, are slightly low, but are quite close to those expected in healthy adults using a similar input device (0.92 to 1.93 bits/s) [20,21]. This result could lead us to conclude that, as head movements are not usually classified as fine motor skills (mostly related with the synchronization of hands and fingers), but as gross motor skills instead, they are mainly developed among children between 3 and 6 years of age [36]. Therefore, the performance of children older than this age range could be similar to that of healthy adults.

It is important to highlight that, as indicated by the results, the values of *TP* are significantly higher after training (from a statistical point of view) for both a standard mouse and the head mouse. The evolution was more noticeable in the case of the head mouse, as children had no previous experience in the use of this device. However, these differences are not clearly observable for the *MT* and the *IP*. These findings suggest that the *TP* is sensitive and therefore a good metric for measuring and characterizing (not only qualitatively, but also quantitatively) changes in the motor behavior after physical training or therapy focused on the motor control improvement.

The error rate results as a function of *W* and *A* values were selected according to the gold standard [37,38]. As expected, the error rate is higher as *W* decreases and *A* increases, and the mean values of the error rate are clearly higher for the head mouse. In fact, even a small reduction in the *A* value is enough to greatly increase the error rate. Taking this into account, it would be reasonable to adjust *W* and *A* to obtain error rates similar to those of the gold standard. These changes would mean a decrease in the *ID* of the movements, which, predictably, would lead to performance improvements.

Regarding the limitations of the study, it should be noted that both the number of participants and their age range were small. Nevertheless, the sample was broad enough to achieve our goals.

Future research directions should be focused on (1) adjusting *A* and *W* values according to the previous proposal, with the aim of achieving performance improvements, and (2) designing a test similar to the one proposed in this paper, but using only the ENLAZA device to evaluate motor performance and the learning process of children with CP undertaking physical therapy sessions.

## Figures and Tables

**Table 1:** Summary of the test parameters (N: number of conditions, *ID*: index of difficulty, *A-W*: amplitude-width in pixels).

***N*** ***ID***	4 1.88 2.66 3.17 4.09
***A-W* (px)**	256–96 512–96 256–32 512–32
**Trials**	13
**Practice**	3
**Test**	10
